# The effects of auditory spatial training on informational masking release in elderly listeners: a study protocol for a randomized clinical trial

**DOI:** 10.12688/f1000research.18602.2

**Published:** 2019-07-04

**Authors:** Farnoush Jarollahi, Marzieh Amiri, Shohreh Jalaie, Seyyed Jalal Sameni

**Affiliations:** 1 Department of Audiology, School of Rehabilitation Sciences, Iran University of Medical Sciences, Tehran, Iran; 2 Department of Physiotherapy, School of Rehabilitation Sciences, Tehran University of Medical Sciences, Tehran, Iran

**Keywords:** Informational masking, Energetic masking, Elderly, Train, Speech perception in noise

## Abstract

**Background:** Regarding the strong auditory spatial plasticity capability of the central auditory system and the effect of short-term and long-term rehabilitation programs in elderly people, it seems that an auditory spatial training can help this population in informational masking release and better track speech in noisy environments. The main purposes of this study are developing an informational masking measurement test and an auditory spatial training program.

**Protocol:** This study will be conducted in two parts. Part 1: develop and determine the validity of an informational masking measurement test by recruiting two groups of young (n=50) and old (n=50) participants with normal hearing who have no difficulty in understanding speech in noisy environments. Part 2 (clinical trial): two groups of 60-75-year-olds with normal hearing, who complain about difficulty in speech perception in noisy environments, will participate as control and intervention groups to examine the effect of auditory spatial training. Intervention: 15 sessions of auditory spatial training. The informational masking measurement test and Speech, Spatial and Qualities of Hearing Scale will be compared before intervention, immediately after intervention, and five weeks after intervention between the two groups.

**Discussion:** Since auditory training programs do not deal with informational masking release, an auditory spatial training will be designed, aiming to improve hearing in noisy environments for elderly populations.

**Trial registration:** Iranian Registry of Clinical Trials ( IRCT20190118042404N1) on 25 ^th^ February 2019.

## Introduction

Many older adults complain about speech perception in noisy situations. It is clear that poorer speech recognition in the elderly population can occur due to various factors, including peripheral hearing impairment or decline in cognitive capabilities and processing defects at supra threshold levels. It is difficult to determine the exact role of each of these factors in developing speech problems in the elderly ^1^. Most elderly individuals complain about the difficulty in understanding speech in noisy environments, despite having normal hearing thresholds ^2^. The spectro-temporal overlap between target and competing speech, which leads to poor target identification, is called energetic masking ^3^. Energetic masking is caused by physical interactions between target and competing speech ^4, 5^ at the low level of the peripheral auditory system ^6^. Recent research has suggested that when competing signals occur randomly or when there is a high similarity between target and competing signals (for example, when both signals are speech), another type of masking occurs. This type of masking, which occurs in response to uncertainty of the competing signal or similarity between target and competing signals, is called non-energetic or informational masking ^7, 8^. It leads to failure in selecting auditory objects and therefore impairs auditory scene analysis. Generally, in contrast with the energetic masking, which occurs due to the limitations caused by frequency selectivity at peripheral levels, informational masking reflects the processing capacity limitations at central auditory levels ^9^. So, the type of background noise heavily influences the extent of the damage imposed to speech intelligibility ^1^. Generally, hearing problems worsen in noisy environments when the target speech is covered by a competing signal. In this situation, in addition to energetic masking, informational masking also occurs ^10^.

Various studies have indicated that with an increase in age, side-effects of competing noises will increase ^1, 10– 12^. Different studies have revealed that elderly populations, who do not have peripheral auditory impairment, suffer from diminished ability of using acoustic and phonetic signs to separate speech from background noise, compared to young people; therefore, more informational masking occurs in this population ^10, 13^. Since problems with understanding speech will reduce social interactions of the elderly population, it is very important to develop effective auditory rehabilitation programs to prevent their isolation and to improve their quality of life ^1^.

Auditory spatial processing plays an important role in speech recognition in complex noisy environments ^14^, since it enables the listener to differentiate the target signal from competing signals via auditory scene analysis and forming auditory streams ^15^. Based on the results of different studies, it has become clear that the most important sign of informational masking release is spatial separation of target and competing signals ^16– 18^. In addition, it has been shown that auditory spatial processing ability is lower in the elderly with normal hearing than in young people. The reduction of localization accuracy and taking advantage of auditory spatial processing, consequently decreasing binaural processing, are not totally related to impaired hearing thresholds ^19, 20^. Hence, it leads to poorer speech recognition in elderly people with normal hearing in noisy environments ^14^. On the other hand, it has been shown that the elderly population need a higher signal to noise ratio for speech recognition in the presence of noise, compared to young people ^11, 21^. These changes are possibly due to the reduction in the ability of using acoustic and phonetic signs to separate target signals from background noise ^11^. Therefore, in elderly people, without considering the peripheral hearing impairment, the ability to use spatial and non-spatial signs for informational masking release diminishes due to the reduction of cognitive processing abilities ^11, 12^, temporal processing defects ^10^, defects in the connection between hemispheres, and diminished ability to separate simultaneous sounds ^11^.

Current neuroscientific studies have suggested that the central auditory system has a strong neuroplasticity capability for auditory spatial processing ^22, 23^ and since the effect of short-term and long-term auditory rehabilitation programs has been demonstrated in elderly people ^2^, it seems that by providing auditory spatial training, we can aid the elderly population to perform informational masking release, preventing them from missing conversations in noisy environments.

The present study has two parts. The first part of this research will be conducted to develop a test for measuring and evaluating the informational masking. Between different researches on informational masking, coordinate response measure (CRM) sentences appear to be a good option to differentiate between energetic and informational masking ^24^. By using these phrases as the target and competing signals, a high semantic and syntactic similarity occurs, which is an important factor in introducing informational masking ^25^. Therefore, since the Persian version of these sentences are not available, we will prepare them and after that, we will develop a new informational measurement test. The second part of this research will be a clinical trial of an auditory spatial training program in elderly people with normal hearing, which would diminish informational masking. The main hypothesis for the second part of the study is that presenting an auditory spatial training for elderly people would be effective in the improvement of speech recognition in noisy environments by stimulating the centers related to binaural processing.

## Protocol

This is version 2 of this protocol.

### Study design, setting and participants

This research will be conducted in the Audiology Clinic of Rehabilitation School of Iran University of Medical Sciences.

This study consists of two main parts. Part 1: develop and determine an informational masking measurement test and explain its validity characteristics in a test development study, conducted cross-sectionally. The study population will be a group of elderly (60 to 75 years old) and a group of young (20 to 40 years old) people. The young people will be recruited from rehabilitation students of Iran University of Medical Sciences, while elderly people are those referred to the audiology clinics of Iran University of Medical Sciences.

Part 2 (simple randomized clinical trial): the effect of training on informational masking release. This part of study is a simple randomized clinical trial design and patients will be randomly assigned into two groups of control (not receiving auditory spatial training) and intervention (receiving auditory spatial training). The random allocation will be performed based on balanced randomization [1:1] where the allocation will be applied by random number table (those assigned an odd number, control group; those assigned an even number, intervention group). This allocation sequence will be generated by one of the audiology clinic staff of the IUMS who will not have any role in the study. An elderly population, 60–75-years-old, who are referred to the audiology clinics of Iran University of Medical Sciences will be selected. The two groups will be matched for age and gender. Those in the control group will not receive any rehabilitation programs during the study.

*Inclusion criteria (for all participants in the study):* auditory thresholds ≤25dB within the 250–8000Hz frequency range for ensuring the normal pure tone audiogram or normal peripheral hearing, ensuring lack of salient cognitive problems using Mini Mental State Examination (MMSE) ^26^; having diploma or higher degree; right-handedness (using Edinburgh handedness inventory); speaking Farsi and being monolingual; complaint about speech in noise perception difficulties (just for those in part 2 of the study); and normal condition of middle ear function.

*Exclusion criteria (for all participants in the study):* unwillingness for participation in each step of research and not meeting inclusion criteria.

### Study procedures

***Part 1: Developing an informational masking measurement test and determining its validity.*** When studying the informational masking, use of the coordinate response measure (CRM) has been frequently been introduced as one of the most popular speech materials for evaluating informational masking ^24^. In these sentences, the same rigid structure with “Ready [call sign] go to [color] [number] now” format is used. In these sentences, eight call signs, four colors, and eight numbers from 1 to 9 can be used. These sentences will be expressed by speakers of different genders ^24, 25, 27^. In the present study, 256 sentences will be created for each speaker (8*4*8). Sentences will be expressed by eight speakers (four women and four men), providing a total of 2048 sentences. Although CRM stimuli have been initially designed to measure the speech perception in the presence of competing signals, these speech materials provide no contextual information; i.e. predicting the given color or number in the phrases is not possible. This is an important factor in measuring informational masking ^25^.

Since there is no Persian version of these sentences, this research will prepare the sentences and determine their content and face validity and reliability. Then, after selecting the nouns, colors, and numbers used in the sentence, conforming to the main English version, the prepared sentences will be given to experts in this field (audiologists, speech therapists, and linguistics) to determine the content validity. These experts are the academic members of rehabilitation schools of IUMS, Tehran University of Medical Sciences (TUMS) and Shahid Beheshti University of Medical Sciences (SBUMS). These experts will be emailed a questionnaire to score the validity items (see Table S1, Extended data).

After selecting the best pattern matching Persian language, based on the model presented for recording the sentences, all sentences will be recorded in a studio with the eight speakers. In order to record the sentences, all criteria of the English version including the sampling rate of 44.1 kHz and giving 3s to speakers to produce each sentence will be followed. Then all the sentences will be scaled and all the words in CRM will be set such that they occur simultaneously, called coordinate sentences ^25^ where each of the sentences will be filtered using a band pass filter of 80 to 8000 Hz filter. Again, in order to determine the face validity, the recorded sentences will be given to the experts mentioned above to determine their suitability. They must fill the questionnaire, which will be emailed to them.

To determine the reliability of CRM speech materials, the mean scores of CRM recognition in the silent will be evaluated in a group of young and old people with normal peripheral hearing who do not have speech perception difficulties in noisy environments. There will be one preliminary test and then a re-test. This evaluation will be implemented at the comfort hearing level of the participants. The score will be calculated based on the correct recognition percentage of the sentences. A sentence will receive a correct score when the color-number combination recognition is recognized correctly ^25, 27^. In this study, the mean correct score for color, number, and noun will be studied separately in order to calculate the error percentage for each of them ^24^. By preparing these sentences, they can be used in the part 2 of the study.

The best way to measure informational masking score is determining the score for speech recognition in the presence of meaningful and meaningless competing noise. To this end, the recognition score for Persian version of CRM speech corpus will be measured under two conditions:

- **A.** *In the presence of meaningful competing noise:* The competing signal will be selected from the Persian version of CRM corpus, where the call sign, color and number used in the competing sentence will be different from those of the target sentence and it will be expressed by a different speaker. The individual will be trained to pay attention to certain target call signs and ignore other signals ^24, 25, 27^. As one of the important effective factors for informational masking is the great similarity between target and competing signals (like when both of them are speech) ^7, 8^, using CRM sentences as both the target signal and competing signal, the high semantic and syntactic similarity would develop between target and competing signals ^24, 25^.
**B.** *In the presence of meaningless competing signal:* the previous signal will be manipulated such that its spectrum content remains fixed but meaningless - indeed, energetic masking will remain but informational masking will be reduced. "Time-reversed speech" will be used for this purpose. This is one of the most effective methods in behavioral and neurophysiological research performed for the effects of speech signals on each other. In this method, by fixing the long-term acoustic spectra of two signals and manipulating one of them such that it divides into non-overlapping time segments, and with reverse time presentation of each segment connecting them to each other, we will have a signal which is equal with the first signal in terms of the spectrum but is not understandable ^28^. In the case of using 20–40 millisecond time windows, this method does not have significant effectiveness in non-understanding the speech signal; therefore, longer time windows should be used ^28^. MATLAB R2018 software will be used in constructing this signal.

The intensity setup of the speech signals with two competing talkers used were similar to some previous studies ^29^. The overall level (RMS power) of target CRM was fixed at the 60 dBSPL. The overall level (RMS power) of each masker was adjusted relative to the target’s level to produce one target-to-masker ratios (TMRs). Initially the masker level was varied in 4 dB steps and then they varied adaptively in 2 dB steps. The two CRM masker phrases always had the same RMS power. The TMRs in sentence recognition test in steps A and B was ±8, ±4, and 0. The target signal is always presented from a loudspeaker in the 0-azimuth degree and two competing signals from the loudspeakers, which are at ±45 and ±90 degree and 0 azimuth degree (once with spatial separation and twice in the direction of target signal), where once the competing signal has the same gender as the target signal and another time has a different gender. As a result, 30 conditions will be evaluated at each step (5 TMRs and three spatial angles with two different genders).

Finally, the informational masking score in all 30 conditions will be calculated as follows:

Speech recognition score in the meaningful competing noise condition-speech recognition score at non-understandable noise condition=informational masking score (percentage).

Jakien and Gallun provided mathematical equations by which the effects of age can be predicted for 45 degrees of separation ^30^. We will develop similar equations, compare them with the published equations, and will use these normative functions to assess improvements in performance after training.

In this step of the research, construct validity will be used to determine the validity of the test. For this purpose, Speech, Spatial, and Qualities of Hearing Scale (SSQ) questionnaire score of each individual will be compared against the informational masking score ^31^. Figure S1 (Extended data) represents the participant’s timeline of the first part of the study.

***Part 2: the effect of auditory spatial training on the informational masking release.*** This part of research will be conducted in three steps: before auditory spatial training, during training, and after training.

**1. Assessments prior to auditory spatial training (preliminary interview)**

**-**Obtain patient history to confirm the inclusion and exclusion criteria of the participants
**-**Initial clinical examination, including otoscopy and tympanometry
**-**Perform pure tone audiometry test
**-**Perform MMSE questionnaire to ensure lack of salient cognition problem in the participants ^26^
**-**Determine speech perception difficulties in the presence of noise: this was evaluated with a question: *Do you have difficulty in understanding speech in noisy situations?* There were three response options: yes, no or sometimes. Those who responded yes were entered into the study.
**-**Measure informational masking score using the test constructed in Part 1 (primary outcome)
**-**Measure synthetic sentence identification test (secondary outcome) ^32^
**-**Determine the SSQ score ^31^. The SSQ self-assessment questionnaire will be filled out by the researcher during the preliminary interview. As improving informational masking can improve the speech perception quality of people, this questionnaire will be used to measure the speech perception quality of the participants quantitatively (secondary outcome).
**-** The temporary storage and manipulation of information required to perform a wide range of complex cognitive activities such as learning, and reasoning called working memory. Since the working memory have more influences on the informational masking tasks, so we will measure the ‘Persian Reading Span test’ score in our study ^33^. Participants will asked to read sets of sentences, report on the semantic acceptability of each sentence, and then recall the final word of each sentence (secondary outcome).

**2. Providing auditory spatial training (intervention group only)**

Auditory spatial training is designed based on five signs that are important in informational masking release: angular differentiation between target and competing signals ^16, 34– 36^; signal to noise ratio ^34^; similarity and difference between the target and competing signals ^12^; similar or different gender for target and competing signals ^12, 37^; and meaningfulness of the competing signal ^12^. As one of the main principles of any auditory training program is progression in difficulty so the training sessions will be divided into three general steps by considering the competing signals. In the first step, meaningless competing signals will be used like white noise. In the second step, in order to make the training process somewhat difficult, meaning-carrying signals like speech babble consisting of four speakers will be used. Finally, for making more difficulty, sentence materials with male and female genders will be used. The reason for using the gender factor is that consideration of gender similarity or difference between target and competing signals is one of the signs that adults use for informational masking release ^12, 37^.

In all steps, the target signal will be presented from the loudspeaker at 0-azimuth degree and competing signals from different azimuth angles such as ±45, ±90 and 0 ^38, 39^. Therefore, the difficulty of training will grow in each step by reducing the azimuth angle of the competing signal ^40^.

Sentence signals are selected from Persian version of QuickSIN ^41^. Every step of training is implemented as follows:

The intensity of the competing signal is fixed at 60 dBSPL, and at the beginning the intensity of target signals is 70 dBSPL. Three first sentences will be used for familiarization. If an individual needs more practice, more familiarization sentences will be provided.

An individual will be requested to identify the keywords heard in the target sentences. In the case of true and false identification, required feedback will be provided. If the individual identifies more than 50% of the keywords, the sentence will be considered true. In this signal to noise ratio, 5 sentences will be provided where if the individual identifies more than 50% of the presented sentences, the signal to noise ratio decreases in 5dB steps, after which 5 sentences will be provided again for the individual. If the individual does not have the capability to correctly identify more than 50% of the presented sentences in each signal to noise ratio, the training begins where this signal to noise ratio will be considered as the initial level. The training will continue for 20 minutes and the intensity will change in an adaptive procedure, such that in the case of correct identification of a sentence, the intensity will be decreased by 1.5dB while it will be increased by 2.5 dB if the individual scored less than 50% of words correctly. At each intensity where an individual can correctly identify the sentences, the next sentence will be presented and the above process will repeat.

The optimal condition for perceptual auditory learning includes active listening to high repetition of signals during the consecutive educational sessions, which is conducted within a short time interval. Since long-term training is not a very suitable option in the clinic ^2^, trainings will be repeated three times a week completed in 5-week cycles ^42^.

**3. Assessments after auditory spatial training (interview immediately after and five weeks after training)**

The informational masking test (as per Part 1) will be done immediately after training and five weeks after that using the Persian list of the coordinate response measure (CRM) corpus, which will be compared with the pre-training results (preliminary interview). This score will be the primary outcome. The reason for repeating experiments five weeks after the intervention is determining the reliability of the results obtained by intervention for informational masking release.

The informational masking release value will be calculated based on the difference between sentence recognition score (in all 30 conditions of signal to noise, different spatial angles, and two genders) in both noise situations (meaningful and non-understandable). The changes in informational masking in the assessments will be calculated before and after the intervention across all 30 conditions (see Table S2, Extended data).

The ‘synthetic sentence identification test’ ^32^ and the ‘Persian Reading Span test’ score ^33^.also will be evaluated immediately after training and 5 weeks after that.

As the ultimate purpose of this research is improving the quality of speech perception of elderly people, the score of SSQ immediately and 5 weeks after intervention will be obtained and the results of both intervention and control groups will be compared separately. This score and the scores of ‘synthetic sentence identification test’ and ‘Persian reading span test’ will be the secondary outcomes of the interventions. Figure S2 (Extended data) represents participant’s timeline of the second part of the study.

### Sample size

***Part 1.*** The study of Terwee *et al.* was considered as the basic study to determine the sample size of the first part of our study. They suggested that at least 50 patients in each group must be included to evaluate the construct validity ^43^. In total, 50 young people aged between 20 and 40 years and 50 elderly people aged between 60 and 75 years, with normal peripheral hearing who do not suffer from speech understanding in noisy environments, will be recruited.

***Part 2.*** The following formula is used to determine the sample size:

$$\text{n}=\frac{{\left(Z_{1-\alpha /2}+Z_{1-\beta }\right)}^{2}\left({S_{1}}^{2}+{S_{2}}^{2}\right)}{\left(\mu_{1}-\mu_{2}\right)}$$

n= (Z1−α/2+Z1−β)2(S12+S22)(μ1−μ2)

S _1_: standard deviation of the studied variable in the first group (case, exposed, or intervened)

S _2_: standard deviation of the studied variable in the second group (control, unexposed, or compared)

µ _1_: mean of the studied variable in the first group

µ _2_: mean of the studied variable in the second group

α=0.05

β=80%

Z= 1.96

In this formula, the studied variable is the extent of informational masking changes before and after the intervention. There is no previous study, which was used the same training as this study proposes; therefore, we considered the study of Delphi *et al.* which was on a group of elderly individuals ^44^. In her study the mean and standard deviation of the improvement in the main variable were 37.5 and 25.17 in the experimental group and 25.17 and 18.15 in the control group, respectively. According to this, the sample size calculated 14 in each group. By assuming 20% drop out, so we will use 18 people in each group.

### Data analysis

Central tendency and dispersion indices (mean and standard deviation) will be used in descriptive analysis of data. In data analysis and for determining the reliability of the Persian version of the coordinate response measure (CRM) corpus, paired t-test and Pearson correlation will be used in the case of normality of data; otherwise, Spearman test will be employed, and one-way ANOVA will be utilized for determining intra-class correlation coefficient.

In part 2 of the research, repeated measures ANOVA values will be used for inter-group comparisons and two-way ANOVA will be employed for comparison among groups. SPSS software (V20.0, IBM Corporation, New York, USA) will be used for statistical data analysis and the significance level for all tests will be 0.05.

### Ethical statement and consent to participate

The Medical Ethics Committee of Iran University of medical sciences approved the study protocol (IR.IUMS.REC.1397.303) and the ethical principles of the ethics committee will be observed in this research. Researchers will send any amendments to the protocol in the future to the ethics committee.

One of the researchers of this study will obtain written informed consent from patients willing to participate in the trial (see Extended data). The purpose of the research and its steps will be explained for all participants before the study start. Confidentiality of data and results of tests will be ensured to participants. Participants will be made aware that they can refrain from cooperation in the study when they want. Conducting tests has no side-effects for the studied individuals and all tests and training sessions will be without cost to the participants.

Since this will be the first time of performing this training, we will running a focus group and asking our participants what they will think of the intervention and how likely they will continue performing the auditory training in the future.

To promote participant retention and complete follow-up, in every training session the examiner will provide feedback to all participants and will inform them about the training progress. The researcher will ask them about the impact of training on the participant’s daily communication conversations. Also at the first of each training session the initial level of training will be calculated and if there will be a progress in the initial signal to noise ratio which train will be started from that, the examiner will inform the patient.

All data will be entered into forms which are prepared for data collection (see Table S2; Extended data) and the participant files will be stored at study site and will be maintained in a secure place and manner. Participant files will be maintained in storage for a period of 2 years after completion of the study. Only Principal Investigators will be given access to the study data.

### Dissemination

The study outcomes will be published through peer-reviewed journals. The data resulting from this study will be released to the audiologists and participants and the general medical community. The results of this trial will be communicated to the external funding body through a formal report. There is no limit in the publication of the trial results.

### Study status

The study started in December 2018 and will continue until December 2019. To date, the enrolment of the patients has been performed and the allocation will be performed in the near future.

## Discussion

Since there is a progressive increase in elderly populations around the world, the independence of this age group has gained much attention, and Iran is no exception ^45^. One of the most important points in independent life during aging is the capability for effective verbal communication. Unfortunately, this capability declines in elderly people, especially tracking speech in environments where several speakers talk with each other. Most elderly individuals complain that despite good hearing, they cannot understand speech in noisy environments ^1^. Indeed, elderly people cannot use auditory spatial signs for informational masking release due to the reduction of their auditory processing and cognitive abilities ^11, 12^. Since informational masking has an important role in competing signal environments and rehabilitation programs have not considered this an important aspect of masking, designing training that can help elderly people in releasing this masking is novel. Therefore, if the main research hypothesis, i.e. auditory spatial training can improve informational masking release in the elderly people, is confirmed, by providing the therapeutic solution in this age group, a series of auditory spatial trainings based on informational masking release will be provided for audiologists. In addition, the Persian version of the coordinate response measure (CRM) corpus and its reliability and validity will be calculated to be used in research on speech recognition in noisy environments.

## Data availability

### Underlying data

No data is associated with this article.

### Extended data

Open Science Framework: The effects of auditory spatial training on informational masking release in elderly listeners: a study protocol for a randomized clinical trial. https://doi.org/10.17605/OSF.IO/SDEJP ^46^.

This project contains the following extended data:

- Table S1: The questionnaire of content and face validity of Persian version of coordinate response measure (CRM) corpus
- Table S2: The data collection sheet for the second part of the study
- Figure S1: Participant timeline for part 1 of the study (Developing an informational masking measurement test and determining its validity)
- Figure S2: Participant timeline for part 2 of the study (the effect of auditory spatial training on the informational masking release)
- Informed consent form for the participants of the first part of the study
- Informed consent form for the participants of the second part of the study

Data are available under the terms of the Creative Commons Zero "No rights reserved" data waiver (CC0 1.0 Public domain dedication).

### Reporting guidelines

Open Science Framework: SPIRIT checklist for ‘The effects of auditory spatial training on informational masking release in elderly listeners: a study protocol for a randomized clinical trial’: https://doi.org/10.17605/OSF.IO/SDEJP ^46^.

## References

[1] Goossens T Vercammen C Wouters J: Masked speech perception across the adult lifespan: Impact of age and hearing impairment. *Hear Res.* 2017;344:109–124. 10.1016/j.heares.2016.11.004 27845259

[2] Anderson S Kraus N: Auditory Training: Evidence for Neural Plasticity in Older Adults. *Perspect Hear Hear Disord Res Res Diagn.* 2013;17:37–57. 10.1044/hhd17.1.37 25485037 PMC4254805

[3] Dai B McQueen JM Hagoort P: Pure linguistic interference during comprehension of competing speech signals. *J Acoust Soc Am.* 2017;141(3):EL249. 10.1121/1.4977590 28372048

[4] Lidestam B Holgersson J Moradi S: Comparison of informational vs. energetic masking effects on speechreading performance. *Front Psychol.* 2014;5:639. 10.3389/fpsyg.2014.00639 25009520 PMC4068195

[5] Pollack I: Auditory informational masking. *J Acoust Soc Am.* 1975;57 10.1121/1.1995329

[6] Newman RS Morini G Ahsan F: Linguistically-based informational masking in preschool children. *J Acoust Soc Am.* 2015;138(1):EL93–8. 10.1121/1.4921677 26233069 PMC4506292

[7] Wightman FL Kistler DJ O'Bryan A: Individual differences and age effects in a dichotic informational masking paradigm. *J Acoust Soc Am.* 2010;128(1):270–9. 10.1121/1.3436536 20649222 PMC2921429

[8] Durlach NI Mason CR Kidd G Jr: Note on informational masking. *J Acoust Soc Am.* 2003;113(6):2984–7. 10.1121/1.1570435 12822768

[9] Calcus A Colin C Deltenre P: Informational masking of speech in dyslexic children. *J Acoust Soc Am.* 2015;137(6):El496–502. 10.1121/1.4922012 26093461

[10] Strouse A Ashmead DH Ohde RN: Temporal processing in the aging auditory system. *J Acoust Soc Am.* 1998;104(4):2385–99. 10.1121/1.423748 10491702

[11] Rajan R Cainer KE: Ageing without hearing loss or cognitive impairment causes a decrease in speech intelligibility only in informational maskers. *Neuroscience.* 2008;154(2):784–95. 10.1016/j.neuroscience.2008.03.067 18485606

[12] Lu Z Daneman M Schneider BA: Does increasing the intelligibility of a competing sound source interfere more with speech comprehension in older adults than it does in younger adults? *Atten Percept Psychophys.* 2016;78(8):2655–2677. 10.3758/s13414-016-1193-5 27566326

[13] Micheyl C Arthaud P Reinhart C: Informational masking in normal-hearing and hearing-impaired listeners. *Acta Otolaryngol.* 2000;120(2):242–6. 10.1080/000164800750001017 11603782

[14] Glyde H Hickson L Cameron S: Problems hearing in noise in older adults: a review of spatial processing disorder. *Trends Amplif.* 2011;15(3):116–26. 10.1177/1084713811424885 22072599 PMC4040826

[15] Ahveninen J Kopčo N Jääskeläinen IP: Psychophysics and neuronal bases of sound localization in humans. *Hear Res.* 2014;307:86–97. 10.1016/j.heares.2013.07.008 23886698 PMC3858499

[16] Yost WA: Spatial release from masking based on binaural processing for up to six maskers. *J Acoust Soc Am.* 2017;141(3):2093–2106. 10.1121/1.4978614 28372135 PMC5848840

[17] Freyman RL Helfer KS McCall DD: The role of perceived spatial separation in the unmasking of speech. *J Acoust Soc Am.* 1999;106(6):3578–88. 10.1121/1.428211 10615698

[18] Litovsky RY: Spatial release from masking. *Acoust.* 2012;8:18–25. Reference Source

[19] Dubno JR Ahlstrom JB Horwitz AR: Binaural advantage for younger and older adults with normal hearing. *J Speech Lang Hear Res.* 2008;51(2):539–56. 10.1044/1092-4388(2008/039) 18367695

[20] Glyde H Buchholz JM Dillon H: The importance of interaural time differences and level differences in spatial release from masking. *J Acoust Soc Am.* 2013;134(2):El147–52. 10.1121/1.4812441 23927217

[21] Shojaei E Ashayeri H Jafari Z: Effect of signal to noise ratio on the speech perception ability of older adults. *Med J Islam Repub Iran.* 2016;30:342. 27390712 PMC4898833

[22] Shinn-Cunningham B: Models of plasticity in spatial auditory processing. *Audiol Neurootol.* 2001;6(4):187–191. 10.1159/000046830 11694725

[23] Sweetow RW Sabes JH: The need for and development of an adaptive Listening and Communication Enhancement (LACE) Program. *J Am Acad Audiol.* 2006;17(8):538–58. 10.3766/jaaa.17.8.2 16999250

[24] Brungart DS: Informational and energetic masking effects in the perception of two simultaneous talkers. *J Acoust Soc Am.* 2001;109(3):1101–9. 10.1121/1.1345696 11303924

[25] Brungart DS Simpson BD Ericson MA: Informational and energetic masking effects in the perception of multiple simultaneous talkers. *J Acoust Soc Am.* 2001;110(5 Pt 1):2527–38. 10.1121/1.1408946 11757942

[26] Ansari NN Naghdi S Hasson S: Validation of a Mini-Mental State Examination (MMSE) for the Persian population: a pilot study. *Appl Neuropsychol.* 2010;17(3):190–5. 10.1080/09084282.2010.499773 20799110

[27] Bolia RS Nelson WT Ericson MA: A speech corpus for multitalker communications research. *J Acoust Soc Am.* 2000;107(2):1065–6. 10.1121/1.428288 10687719

[28] Ueda K Nakajima Y Ellermeier W: Intelligibility of locally time-reversed speech: A multilingual comparison. *Sci Rep.* 2017;7(1): 1782. 10.1038/s41598-017-01831-z PMC5431844 28496124

[29] Marrone N Mason CR Kidd G Jr: The effects of hearing loss and age on the benefit of spatial separation between multiple talkers in reverberant rooms. *J Acoust Soc Am.* 2008;124(5):3064–75. 10.1121/1.2980441 19045792 PMC2736722

[30] Jakien KM Gallun FJ: Normative Data for a Rapid, Automated Test of Spatial Release From Masking. *Am J Audiol.* 2018;27(4):529–38. 10.1044/2018_AJA-17-0069 30458523 PMC6436452

[31] Gatehouse S Noble W: The Speech, Spatial and Qualities of Hearing Scale (SSQ). *Int J Audiol.* 2004;43(2):85–99. 10.1080/14992020400050014 15035561 PMC5593096

[32] Rahbar N Kamali M Pourgharib J: Development And Evaluation A Farsi Language Version Of Synthetic Sentence Identification Test In Normal Individuals. *Auditory and Vestibular Research.* 2006;15(1):27–31. Reference Source

[33] Shahnazari M: The development of a Persian reading span test for the measure of L1 Persian EFL learners’ working memory capacity. *J Applied Research on English Language.* 2013;2(2):107–16. 10.22108/are.2013.15473

[34] Glyde H Buchholz J Dillon H: The effect of better-ear glimpsing on spatial release from masking. *J Acoust Soc Am.* 2013;134(4):2937–45. 10.1121/1.4817930 24116429

[35] Lin G Carlile S: Costs of switching auditory spatial attention in following conversational turn-taking. *Front Neurosci.* 2015;9:124. 10.3389/fnins.2015.00124 25941466 PMC4403343

[36] Freyman RL Balakrishnan U Helfer KS: Effect of number of masking talkers and auditory priming on informational masking in speech recognition. *J Acoust Soc Am.* 2004;115(5 Pt 1):2246–56. 10.1121/1.1689343 15139635

[37] Brungart DS Simpson BD: The effects of spatial separation in distance on the informational and energetic masking of a nearby speech signal. *J Acoust Soc Am.* 2002;112(2):664–76. 10.1121/1.1490592 12186046

[38] Glyde H Cameron S Dillon H: Remediation of spatial processing deficits in hearing-impaired children and adults. *J Am Acad Audiol.* 2014;25(6):549–61. 10.3766/jaaa.25.6.5 25313545

[39] Cameron S Glyde H Dillon H: Efficacy of the LiSN & Learn auditory training software: randomized blinded controlled study. *Audiol Res.* 2012;2(1):e15. 10.4081/audiores.2012.e15 26557330 PMC4630948

[40] Tyler RS Witt SA Dunn CC: Initial development of a spatially separated speech-in-noise and localization training program. *J Am Acad Audiol.* 2010;21(6):390–403. 10.3766/jaaa.21.6.4 20701836 PMC2947843

[41] Shayanmehr S Tahaei AA Fatahi J: Development, validity and reliability of Persian quick speech in noise test with steady noise. *Aud Vest Res.* 2015;24(4):234–244. Reference Source

[42] Humes LE Kinney DL Brown SE: The effects of dosage and duration of auditory training for older adults with hearing impairment. *J Acoust Soc Am.* 2014;136(3):EL224. 10.1121/1.4890663 25190425 PMC4144170

[43] Terwee CB Bot SD de Boer MR: Quality criteria were proposed for measurement properties of health status questionnaires. *J Clin Epidemiol.* 2007;60(1):34–42. 10.1016/j.jclinepi.2006.03.012 17161752

[44] Delphi M Lotfi Y Moossavi A: Envelope-based inter-aural time difference localization training to improve speech-in-noise perception in the elderly. *Med J Islam Repub Iran.* 2017;31:36. 10.14196/mjiri.31.36 29445665 PMC5804443

[45] Sadoughi F Shahi M Ahmadi M: The Comparison of the Minimum Data Set for Elderly Health in Selected Countries. *Acta Inform Med.* 2015;23(6):393–397. 10.5455/aim.2015.23.393-397 26862252 PMC4720821

[46] Amiri M: The Effects of Auditory Spatial Training on Informational Masking Release in Elderly Listeners: A Study Protocol for a Randomized Clinical Trial.OSF.2019 10.17605/OSF.IO/SDEJP PMC6652096 31354946

