# Supplementary material for: The effects of auditory spatial training on informational masking release in elderly listeners: a study protocol for a randomized clinical trial
Source: F1000Res. 2019 Jul 4;8:420. Originally published 2019 Apr 9. [Version 2] doi: 10.12688/f1000research.18602.2 (PMC6652096; doi:10.12688/f1000research.18602.2)
Supplement: Peer review history

# Peer review: Reviewer response for version 2

**DOI:** 10.5256/f1000research.21665.r50797

**Published:** 2019-07-22

**Authors:** Frederick J. Gallun, Aaron Seitz

**Affiliations:** 1 Department of Psychology, University of California, Riverside, USA; 2 VA RR&D, National Center for Rehabilitative Auditory Research (NCRAR), VA Portland Health Care System (VAPORHCS), Portland, OR, USA; 3 Dept. of Otolaryngology/Head & Neck Surgery, Oregon Health and Science University, Portland, USA

Authors have addressed all concerns.

We confirm that we have read this submission and believe that we have an appropriate level of expertise to confirm that it is of an acceptable scientific standard.

# Peer review: Reviewer response for version 2

**DOI:** 10.5256/f1000research.21665.r50796

**Published:** 2019-07-16

**Authors:** Nehzat Koohi

**Affiliations:** 1 UCL Ear Institute, University College London, London, UK

Most comments are appropriately addressed by the authors.

 Please see minor comments below:

1. The aims in the last paragraph of introduction should be revised, it is not very clear that the target population is elderly people with normal pure tone audiogram who complain of speech in noise difficulties. The title should be revised too:'... elderly listeners with speech in noise difficulty.."
2. Citation is needed for the following sentence: 'In addition, it has been shown that auditory spatial processing ability is lower in the elderly with normal hearing than in young people.'
3. 'Normal hearing' in the text should be changed to 'normal pure tone audiogram'. 
4. 'auditory thresholds ≤25dB within the 250–8000Hz frequency range for ensuring the normal pure tone audiogram' as per ASHA protocol?
5. 'normal peripheral hearing' in the inclusion criteria section should be removed.
6. 'complaint about speech in noise perception difficulties' as judged by SSQ?

I confirm that I have read this submission and believe that I have an appropriate level of expertise to confirm that it is of an acceptable scientific standard.

# Peer review: Reviewer response for version 1

**DOI:** 10.5256/f1000research.20365.r46974

**Published:** 2019-05-20

**Authors:** Nehzat Koohi

**Affiliations:** 1 UCL Ear Institute, University College London, London, UK

**This study has two aims: first developing and validating a test to measure informational masking and second to conduct a randomised clinical trial to evaluate the effectiveness of an auditory training in elderly people who have speech in noise difficulty. The introduction is well-written, and the aims are clearly stated however the methodology needs to be revised. The detailed comments are as below:**

**Introduction**

First Paragraph

- ‘Understanding speech in noisy environments is a major challenge of the auditory system, which occurs mostly due to aging.’ Please revise this sentence. There are many reasons for difficulty understanding speech in background noise, aging is one of them, it doesn’t occur mostly due to aging.
- Please first define/explain what energetic and informational masking are (please re-organise your paragraphs, the definition should come first)

 

Second paragraph

- ‘…, another type of masking occurs both.’ Please revise this sentence, it doesn’t make sense.

 

Fourth paragraph

- ‘Based on the results of different studies, it has become clear that the most important sign of informational masking release is spatial separation of target and competing signals’. Here you’ve mentioned several studies but only used one reference, either revise the sentence or add more references.

 

Fifth paragraph

- ‘On the other hand, it has been shown that the elderly population need a higher signal to noise ratio for speech recognition in the presence of noise, compared to young people.’ Please add a reference.
- ‘in elderly people, without considering the hearing impairment, the ability to use spatial and non-spatial signs for informational masking release diminishes due to the reduction of cognitive processing abilities, temporal processing, defects in the connection between hemispheres, and diminished ability to separate simultaneous sounds.’ By ‘hearing impairment’ do you mean apparent hearing loss on the PTA? Hearing impairment includes temporal (and auditory) processing deficits as well as peripheral hearing impairment.

 

Last paragraph

- ‘The present study had two parts. The first part of this research was developing a test for measuring and evaluating the informational masking.’ The sentences are in the past, have you done the study already or you are going to conduct these? In the study design section, you mention ‘the study will be conducted...’ Please be consistent. 
- Although you have clarified this in your methodology, your aim will be clearer if you specify here why you are developing and validating a test to measure informational masking.

 

**Study design**

- ‘Inclusion criteria (for all participants in the study): auditory thresholds ≤25dB within the 250–2000Hz frequency range and …’ My main concern is the definition of ‘normal hearing’. Which protocol have you used to define ‘normal hearing’? if you are including patients with mild high frequency hearing loss you cannot say patient’s hearing is normal. Even if all frequencies (250-8000 Hz) are better than 20 or 25 dBHL (BSA or ASHA definition) if the OAEs are not robust or wave I of the ABR is absent you cannot assume patient’s hearing is normal. Pure tone audiogram is very limited when testing the auditory pathway. In addition, you need to differentiate between peripheral and central hearing. If you would like to use the term normal hearing, it’s better to say ‘peripheral hearing’ as you have not tested the central auditory pathway so you cannot assume their hearing is ‘normal’. The prevalence of hearing impairment (peripheral and central) in elderly people is quite high. Also it is perfectly possible that an elderly patient has a spatial processing disorder in the presence of 'normal pure tone audiogram'.
- SSQ is not a quality of life questionnaire, please use another term and revise this paragraph.
- Please justify why you chose 4 speakers.

  

**Sample size**

- Please specify what Z in the sample size formula is and include means and standard deviations from the Delphi’s study. The sample size for study 2 is not adequately justified. Sixteen participants seems a very low number, particularly if we assume 20% drop out. How many participants are you going to approach?  

  

**Other comments**

- Sufficient time must be considered for auditory training, one month seems a very short time to assume auditory training is beneficial or not (in Humes et al’s methodology the training was performed twice a day for 7.5 weeks but you are proposing 2 trainings in 4 weeks) , ideally you should repeat your outcome measures after 3 and 6 months, and even better if you could repeat 1 month after the training is ended to explore the long term potentiation. Feedback and monitoring are crucial in auditory training, you have mentioned about the feedback but please explain in detail how this will be done. There was no mention of progression in difficulty of the training (one of the main principles of auditory training), are you going to consider this in your training?
- Since you have not done any pilot or feasibility study, it would be useful if you could do a small qualitative study; for example, running a focus group and asking your participants what they think of the intervention and how likely they will continue performing the auditory training in the future. One of the major issues with the auditory training (or any other training) is boredom and generally keeping the patient motivated throughout. In addition, in practice, performing the training in the clinic is time consuming and costly. Would patients do these training at home? Adhering to the training while performing at home is another issue.
- The consent form needs to be written in lay language and please avoid jargon and acronyms. 

I confirm that I have read this submission and believe that I have an appropriate level of expertise to confirm that it is of an acceptable scientific standard, however I have significant reservations, as outlined above.

# Response

**Published:** 2019-07-07

**Authors:** Marzieh Amiri

**Affiliations:** Iran University of Medical Sciences, Iran

Dear Dr, Koohi

We appreciate you for your complimentary comments and suggestions. The followings are our point-by-point responses:

1 Your comments about ‘ **Introduction**’ part were:

**First paragraph:** please revise this sentence:’ Understanding speech in noisy environments is a major challenge of the auditory system, which occurs mostly due to aging.’ And please first define/explain what energetic and informational masking are (please re-organize your paragraphs, the definition should come first)

**Response**: As suggested by you, we have revised the first sentence to:

‘Many older adults complain about speech perception in noisy situations.’  And we also re-organize the paragraph according to your comment.

**Second paragraph**:  Another type of masking occurs both.’ Please revise this sentence, it doesn’t make sense.

**Response**: We are sorry that this part was not clear in the original manuscript. We have revised the contents of this part.

**Fourth paragraph: **‘Based on the results of different studies, it has become clear that the most important sign of informational masking release is spatial separation of target and competing signals’. Here you’ve mentioned several studies but only used one reference, either revise the sentence or add more references.

**Response**: we have added more references.

**Fifth paragraph: **‘On the other hand, it has been shown that the elderly population need a higher signal to noise ratio for speech recognition in the presence of noise, compared to young people.’ Please add a reference.

**Response** **: we have added references.**

‘in elderly people, without considering the hearing impairment, the ability to use spatial and non-spatial signs for informational masking release diminishes due to the reduction of cognitive processing abilities, temporal processing, defects in the connection between hemispheres, and diminished ability to separate simultaneous sounds.’ By ‘hearing impairment’ do you mean apparent hearing loss on the PTA? Hearing impairment includes temporal (and auditory) processing deficits as well as peripheral hearing impairment.

**Response**: Your comment is totally true. We have mentioned ‘peripheral hearing impairment’ in the sentences.

**Last paragraph: **‘The present study had two parts. The first part of this research was developing a test for measuring and evaluating the informational masking.’ The sentences are in the past, have you done the study already or you are going to conduct these? In the study design section, you mention ‘the study will be conducted...’ Please be consistent. 

**Response: **We have corrected the sentences.

Although you have clarified this in your methodology, your aim will be clearer if you specify here why you are developing and validating a test to measure informational masking.

**Response: **We have explained this and added more sentences to this part.

2 Your comments about the ‘ **study design’:**

‘ **Inclusion criteria**: You need to differentiate between peripheral and central hearing. If you would like to use the term normal hearing, it’s better to say ‘peripheral hearing’ as you have not tested the central auditory pathway so you cannot assume their hearing is ‘normal’.

**Response: **We have revised this criteria accordingly to: ****

“Auditory thresholds ≤25dB within the 250–8000Hz frequency range for ensuring the normal pure tone audiogram or normal peripheral hearing,”

**SSQ** is not a quality of life questionnaire, please use another term and revise this paragraph.

**Response:**  We have revised this sentence as bellow:

‘As improving informational masking can improve the speech perception quality of people, this questionnaire will be used to measure the speech perception quality of the participants quantitatively (secondary outcome).’

**Please justify why you chose 4 speakers.**

**Response: **We have corrected this part and we will use eight speakers for recording the CRM phrases.

3 Your comments about the ‘ **sample size:**

Please specify what Z in the sample size formula is and include means and standard deviations from the Delphi’s study. The sample size for study 2 is not adequately justified. Sixteen participants seems a very low number, particularly if we assume 20% drop out. How many participants are you going to approach?  

**Response: **We have included all you mentioned in the revised version.

4 **Other comments** you mentioned were:

1) **Training period** which have corrected as bellow

‘The optimal condition for perceptual auditory learning includes active listening to high repetition of signals during the consecutive educational sessions, which is conducted within a short time interval. Since long-term training is not a very suitable option in the, trainings are repeated three times a week completed in 5-week cycles .’

2 **) Feedback and monitoring** are crucial in auditory training, you have mentioned about the feedback but please explain in detail how this will be done.

**Response:** **We have included your comment to the text.**

3)  There was no mention of **progression** in difficulty of the training (one of the main principles of auditory training), are you going to consider this in your training?

**Response: **We have added more explanation to this part. As bellow.

**‘**As one of the main principles of any auditory training program is progression in difficulty so the training sessions will be divided into three general steps by considering the competing signals. In the first step, meaningless competing signals will be used like white noise. In the second step, in order to make the training process somewhat difficult, meaning-carrying signals like speech babble consisting of four speakers will be used. Finally, for making more difficulty, sentence materials with male and female genders will be used. The reason for using the gender factor is that consideration of gender similarity or difference between target and competing signals is one of the signs that adults use for informational masking release.’

4) Since you have not done any pilot or feasibility study, it would be useful if you could do a small qualitative study; for example, running a focus group and asking your participants what they think of the intervention and how likely they will continue performing the auditory training in the future.

**Response:** You have mentioned a very good point and we have added your comment to the text. You can find it in the third paragraph of the ‘Ethical statement and consent to participate’ part.

5) Would patients do these training at home?

**Response:  **Because we will design our study with loudspeakers it will be not done at home. But this study is kind of a pilot study and if this training will have good results maybe it would be done in the future and under headphones.

6) The **consent form** needs to be written in lay language and please avoid jargon and acronyms. 

**Response:** **We have revised it.**

# Peer review: Reviewer response for version 1

**DOI:** 10.5256/f1000research.20365.r47087

**Published:** 2019-04-23

**Authors:** Frederick J. Gallun, Aaron Seitz

**Affiliations:** 1 VA RR&D, National Center for Rehabilitative Auditory Research (NCRAR), VA Portland Health Care System (VAPORHCS), Portland, OR, USA; 2 Dept. of Otolaryngology/Head & Neck Surgery, Oregon Health and Science University, Portland, USA; 3 Department of Psychology, University of California, Riverside, USA

Overall, we think it is an interesting study and the goal of creating a Persian version of the CRM and spatial release tests is a good idea. However, the methods for developing the new test create opportunities for confounds and difficulties interpreting the data. Specific comments are listed below. In addition, the training arm is not sufficiently different from the new informational masking for it to be a clinically reliable outcome measure. It would be appropriate to have another task (such as dichotic sentence identification (or equivalent test that has already been validated in the Persian language)) that wasn't trained as an outcome. Furthermore, the lack of an active control condition raises the possibility that any activity involving remembering and responding to stimuli (or even just coming into the testing environment) would change performance. For detailed discussion of the training issues, the reader is encouraged to consult Green et al. (2019) ^1^. Additional fundamental methodological concerns involve the failure to consider the working memory and attention influences that are known to be important for speech in noise for older listeners (Fullgrabe et al., 2015) ^2^ and for all listeners for informational masking tasks, especially the CRM tasks.

 With the regard to the new CRM test, it is excellent to create a Persian version, but there are several differences in these methods that could result in substantial differences in the outcomes.

 The step size of 5 dB is too large, given the differences in performance that are usually observed. It might be acceptable if a psychometric function were being fit to the data, but the statistical analysis proposed is unlikely to be sufficiently sensitive to the small changes that this method is able to detect, using the standard methods. A more appropriate measure is the target-to-masker ratio at which a fixed level of performance is obtained. For examples of the differences in target-to-masker ratio commonly observed in older and younger listeners with the English version for same and different-gender targets and maskers, the reader is directed to Marrone et al. (2008b) ^3^ and Gallun et al. (2013) ^4^. 

 The use of two male and two female speakers is not sufficient to ensure that the specific speaking styles of the talkers are not influencing the results. English CRM uses four of each gender, and the studies from our lab exclude one of the males due to differences in rate of speaking. Time alignment of the keywords is an essential aspect of creating an informational masking situation where spatial cues can provide large release from masking. Temporal overlap should be carefully examined, and preliminary testing should establish that none of the talkers is more intelligible than the others in the conditions to be tested. 

 The use of 90 degrees of spatial separation is large enough that it is possible that changes in spatial ability will not be detected. Multiple studies have established that the difference between 30 and 45 degrees of separation is not very large (Marrone et al., (2008a) ^5^; Jakien et al., (2017) ^6^) and that for people with normal hearing, the effects of age are difficult to detect with separations greater than 15 degrees (Srinivasan et al., (2016) ^7^). For this study, this is very important, because if the effects of the training are to improve the ability to use (or even perceive) spatial differences, it is unlikely that this will be a very large change, and so if only very large separations are used, there may be no way to observe the improvements in performance. Jakien and Gallun (2018) ^8^ provided mathematical equations by which the effects of age can be predicted for 45 degrees of separation. It would be useful to develop similar equations in Aim 1, compare them with the published equations, and use these normative functions to assess improvements in performance after training.

 This report was written by Frederick Gallun with advice from Aaron Seitz.

We confirm that we have read this submission and believe that we have an appropriate level of expertise to confirm that it is of an acceptable scientific standard, however we have significant reservations, as outlined above.

## References

[1] : Improving Methodological Standards in Behavioral Interventions for Cognitive Enhancement. *Journal of Cognitive Enhancement*.2019;3(1) : 10.1007/s41465-018-0115-y 2-29 10.1007/s41465-018-0115-y

[2] : Age-group differences in speech identification despite matched audiometrically normal hearing: contributions from auditory temporal processing and cognition. *Front Aging Neurosci*.2014;6: 10.3389/fnagi.2014.00347 347 10.3389/fnagi.2014.00347 25628563PMC4292733

[3] : The effects of hearing loss and age on the benefit of spatial separation between multiple talkers in reverberant rooms. *J Acoust Soc Am*.2008;124(5) : 10.1121/1.2980441 3064-75 10.1121/1.2980441 19045792PMC2736722

[4] : Independent impacts of age and hearing loss on spatial release in a complex auditory environment. *Front Neurosci*.2013;7: 10.3389/fnins.2013.00252 252 10.3389/fnins.2013.00252 24391535PMC3870327

[5] : Tuning in the spatial dimension: evidence from a masked speech identification task. *J Acoust Soc Am*.2008;124(2) : 10.1121/1.2945710 1146-58 10.1121/1.2945710 18681603PMC2809679

[6] : Validating a Rapid, Automated Test of Spatial Release From Masking. *Am J Audiol*.2017;26(4) : 10.1044/2017_AJA-17-0013 507-518 10.1044/2017_AJA-17-0013 28973106PMC5968328

[7] : Release from masking for small spatial separations: Effects of age and hearing loss. *J Acoust Soc Am*.140(1) : 10.1121/1.4954386 EL73 10.1121/1.4954386 27475216PMC5392088

[8] : Normative Data for a Rapid, Automated Test of Spatial Release From Masking. *Am J Audiol*.2018;27(4) : 10.1044/2018_AJA-17-0069 529-538 10.1044/2018_AJA-17-0069 30458523PMC6436452

# Response

**Published:** 2019-07-07

**Authors:** Marzieh Amiri

**Affiliations:** Iran University of Medical Sciences, Iran

**Reviewer 1**

Dear Dr, Gallun,

We appreciate you for your complimentary comments and suggestions. The followings are our point-by-point responses:

1 Your concerns about CRM were as bellow: 1) The **step size** and **use a target-to-masker ratio**.

**Response: **We have revised this part according to Marrone et al.’s study. You can find it in the revised manuscript (Study procedure, Part1, the last paragraph).

**2) The number of speakers:**

**Response: **you are totally right and we will use eight speakers for recording the CRM phrases and we have revised that in the manuscript. ****3) **The use of 90 degrees of spatial separation is large enough that it is possible that changes in spatial ability will not be detected.**

**Response: **Your comment is very useful. We have added the ±45 degree to the study and we have revised the methodology according to your concerns. We also will use the mathematical equations which you mentioned to evaluate the effects of age.

2 Your concerns about training were as bellow:

1 The training arm is not sufficiently different from the new informational masking for it to be a clinically reliable outcome measure. It would be appropriate **to have another task (such as dichotic sentence identification (or equivalent test that has already been validated in the Persian language))** that wasn't trained as an outcome.

**Response: **We also have added the ‘ Farsi language version of synthetic sentence identification test’ in pre and post- training parts of the study. We must mentioned that the ‘dichotic sentence identification test’ have not been converted to Persian. The original manuscript was revised accordingly.

2 Furthermore, the lack of an active control condition raises the possibility that any activity involving remembering and responding to stimuli (or even just coming into the testing environment) would change performance.

**Response: **This condition is the same for both groups. So we assumed that both of them may learn or remember the tests items. So the differences between them will be diminished. Also as you know the CRM phrases are contextual-free and it is a good point of them. It means that being in the test environment have not any large effect on scoring the test and anyone who participated in this study must just rely on her or his audibility to identify the correct color and number.

3 Failure to consider the **working memory** and attention influences.

**Response: **As you correctly concerned about the working memory effects, we have added a ‘ Persian Reading Span test’ to evaluate the working memory ability of the participants. We will try to match people of both groups according to their scores on this test and the other tests which mentioned in the text in pre and post training situations. We must mentioned that we also use the MMSE questionnaire to include the persons who have no salient cognition impairment.
